# Improved cattle farm classification: leveraging machine learning and linked national datasets

**DOI:** 10.3389/fvets.2025.1517173

**Published:** 2025-02-05

**Authors:** Guy-Alain Schnidrig, Rahel Struchen, Sara Schärrer, Dagmar Heim, Daniela Hadorn, Gertraud Schüpbach-Regula, Giulia Paternoster

**Affiliations:** ^1^Veterinary Public Health Institute, Vetsuisse, University of Bern, Bern, Switzerland; ^2^Graduate School of Cellular and Biomedical Sciences, University of Bern, Bern, Switzerland; ^3^Department of Animal Health and Animal Welfare, Federal Food Safety and Veterinary Office (FSVO), Bern, Switzerland

**Keywords:** machine learning, surveillance, farm topology, movement database, antibiotic use data, cattle

## Abstract

While many countries have registries of livestock farms, it can be challenging to obtain information on their primary production type. For example, for Swiss farms registered as keeping cattle, a distinction can only be made between milk-producing and non-milk-producing farms. The Swiss cattle industry consists of beef and dairy farms, with a strong predominance of small to medium-sized farms. A better differentiation of cattle production types would be beneficial for the planning and evaluation of surveillance programmes for cattle diseases and for the benchmarking antibiotic consumption. The aim of this study was to outline cattle production types of interest and to allow the classification of Swiss cattle farms according to production type in order to optimize surveillance. We collaborated with experts to define the five primary cattle production types: calf fattening, dairy cattle, cattle fattening, rearing cattle and suckler cows. In collaboration with the cantonal Veterinary Offices, we collected production types from 618 reference farms across 14 cantons and defined a total of 24 features by combining information from three national databases. Using farm-level data on milk production, age and sex distribution, cattle breeds, calving, births, slaughter, animal movements and antibiotic use, we trained three different machine learning models capable of classifying the five production types. Among these models, the Random Forest model demonstrated the highest level of performance, achieving an accuracy of 0.914 (95% CI: 0.890, 0.938) and an F1-Score of 0.879 (95% CI: 0.841, 0.913). In conclusion, together with experts, we have outlined five primary production types on cattle farms in Switzerland and developed a model that allows a reproducible, year-to-year classification of cattle farms using national datasets. Our flexible methodology could be adapted to other countries and datasets, enabling veterinary authorities to conduct more efficient and targeted disease surveillance in the future.

## 1 Introduction

Several criteria and methods are used to define and classify farms with livestock, each emphasizing different aspects of farming practices, livestock species, and farm size (1–4). For epidemiological purposes it makes sense to refine these classifications, and to include characteristics of the production system which are relevant to animal health (5). Infectious diseases have a severe impact on the productivity and profitability of cattle farms (6–8). The risks related to infectious diseases differ substantially in different production types within the cattle industry. Another more general threat to animal health is the emergence of antibiotic resistance (9, 10). It has been demonstrated that the over- or misuse is one of the principal factors in the development of antibiotic resistance (11, 12). Antibiotic use varies between animal species, farm management practices, biosecurity, and especially production types (13).

The Swiss cattle industry consists of beef and dairy cattle farms with a predominance of small to medium-sized farms, with a median of 37 cattle per farm (14). The cattle population in Switzerland counts a total of 1.5 million animals (15). The practice of summer grazing is common, with approximately one-third of all cattle being moved on mountain pastures on an annual basis (16). Differentiating cattle farms by their primary production type is adventitious for making sound comparisons of antibiotic consumption, for designing effective disease surveillance and control programmes and for facilitating the resource allocation of the official veterinary services.

However, the available data on production types in Switzerland is limited. The production type available in the animal identification and registration system of Switzerland (Animal Movement Database AMD in conformity with EU Regulation 1760/2000) is only roughly divided into dairy cows, mixed and other cattle. The information is collected by self-declaration, and therefore frequently out of date. The cattle numbers in the agricultural policy information system AGIS are calculated from the AMD and contains thus no further details on production systems. The data of breeding and production organizations are private and not a priori available to the public administration. The Federal Food Safety and Veterinary Office (FSVO) employs data from the mandatory bimonthly bulk milk quality control in the context of cattle disease surveillance programmes for the distinction between milk-delivering and non-milk-delivering farms. In summary, the available data sources don't allow to identify farms with fattening calves, rearing or suckling cows.

Various methods have been developed to improve cattle production types. These include expert-based approaches like such as those used by Sala et al. (5), Smith et al. (17), and more advanced methods such as those developed by Brock et al. (18), which integrate domain specialist expertise with an unsupervised machine learning algorithm known as self-organizing maps (SOMs). Conversely, supervised learning involves training an algorithm using a labeled dataset, where each instance is associated with a specific label or target feature (19). However, acquiring the correct production types can be expensive, time-consuming, or even impossible. In this study we present an approach that uses several aspects of previous methods but employs a supervised machine learning algorithm to classify farms into production types.

The aim of this study was to develop and evaluate a tool to classify cattle farms based on available data on milk production, age and sex distribution, cattle breeds, calving, births, slaughter, animal movements and antibiotic use. The ability of the tool to classify Swiss cattle farms into calf fattening, dairy cattle, cattle fattening, rearing cattle and suckler cows was evaluated with a representative sample of farms with their production type verified by cantonal Veterinary Offices (cVOs). Ultimately, we think that the expanded classification will improve disease surveillance by providing the cVOs with more information for decision making.

## 2 Materials and methods

### 2.1 Production type description using expert knowledge

Based on the production types needed for planning and evaluating surveillance of cattle diseases and antibiotic consumption, we identified and described five primary production types: calf fattening farms (6 weeks−6 month), cattle fattening farms (6 month−2 years), dairy farms, rearing farms, and suckler cow farms. Switzerland has many small and mixed cattle farms, so it is possible for a farm to belong to more than one type, but for the purposes of this study we focused on identifying the predominant production type for each farm, which represents its main activity.

In a first step, the most relevant production types were identified based on existing literature and discussions with appropriate experts. The experts (*n* = 4) were selected based on their experience with the Swiss cattle system and their relevant work experience in both national veterinary authorities and cattle producer associations. An expert survey was then conducted to develop a list of expected characteristics for each production type. The selected experts reviewed and completed the production type description consisting of a list of expected characteristics. Each cattle production type was defined according to these expected characteristics including herd structure in terms of breed (Supplementary Table 1), sex and age, and animal movements. We collected the expected characteristics of the production types to enable comparison with the characteristics in the labeled cantonal data and our classification model.

Production types and their expected characteristics in Switzerland are described in Table 1. Essentially, calf fattening farms do not have commercial milk production and are expected to predominantly handle male cattle aged between 0 and 6 months. They have a low number of births receive inflows from multiple sources and have many animals leaving for slaughter. Dairy farms are characterized by milk production and maintain predominantly female cattle of various ages. They have frequent births and a significant outflow of male calves for fattening or slaughter. Cattle fattening farms have predominantly beef breeds and no commercial milk production. They receive young males aged 6 to 24 months for fattening and slaughter. Rearing cattle farms predominately handle heifers (female cattle aged between 6 and 24 months). With a limited number of births, they are expected to have a high proportion of animals returning to their birth herd. Suckler cow farms maintain a predominantly female cattle herd, including few bulls, across all age ranges and might have commercial milk production. With frequent births due to the continuous cycle of breeding, animals may be slaughtered between 8 and 15 months of age.

**Table 1:** Swiss cattle production types and their expected characteristics based on expert knowledge.

**Production type**	**Has commercial milk**	**Cattle breeds**	**Sex**	**Age**	**Births**	**Calved animals**	**Animal traffic (*exclusive slaughter*)**	**Exits for slaughter**	**Age at slaughter**
Calf fattening	No	Mixed	Mixed to more male	0–6 months	Few to none	None	Predominantly inflows of young animals, few to none leaving, many farms of origin	Yes many	Calves 0–6 months
Dairy cow	Yes	Predominantly dairy	Predominantly female	Cows >24 months, calves 0–6 months, young cattle 6–24 months	Yes many	Yes many	Outgoing bull calves (for fattening or slaughter), incoming young females 24 months old	Yes	Cows >24 months, calves 0–6 months
Cattle fattening	No	Predominantly beef	Mixed to more male	6–24 months	Few to none	Few to none	Predominantly inflows of young animals, few to none leaving, many farms of origin	Yes many	6–24 months
Rearing cattle	No	No specific	Predominantly female	6–24 months	Few to none	Few to none	Many entries/exits, entries of cow cales exits of young females, incoming young females 24 months old	Few to none	6–24 months
Suckler cow	No but can have	Predominantly beef or dual-purpose	Predominantly female, 1 or more bulls	All ranges	Yes many	Yes many	Few to none	Yes	8–15 months

### 2.2 Datasets and feature engineering

We used data from three different national databases to define features representing the previously defined production type characteristics. The three datasets were derived from the AMD, aRes, and IS ABV (definition as follows) and combined by a unique farm identifier (AMD number). **AMD** is the animal movement database of Switzerland. From there we extracted individual animal data including age, sex, breed, calving, slaughter, and movement history. Based on this data, we developed herd and movement characteristics at farm level (Table 2) adapting the work of Brock et al. (18) to the Swiss cattle production system. **aRes** serves as the national database for results of diagnostic tests carried out by laboratories on behalf of the official veterinary services. Based on these laboratory data, we checked whether the farm had at least one result from official milk quality controls in a defined year indicating that the farm had produced commercial milk. **IS ABV** is the database containing all prescriptions of veterinary antibiotics since 2019 (20). We used data from IS ABV to calculate the number of prescriptions per farm in relation to the total number of prescriptions. The datasets were extracted for the years 2020, 2021, and 2022 and after aggregation the AMD number was anonymised to ensure data protection. In Switzerland there are a total of 36,206 farms that had at least one or more cattle (AMD, 2022). In this study we only included cattle farms with a total of at least 10 cattle stays per year, i.e., at least 10 instances of cattle being kept on the farm, regardless of how long they had been on the farm. Additionally, we excluded farms registered as alpine pasture facilities, veterinary clinics, or cattle markets (remaining cattle farms *N* = 34,226). Based on the expected characteristics of cattle production types described in Table 1, we defined 24 features for each farm as described in Table 2.

**Table 2:** Description, calculation, and data source of the 24 features defined for each cattle farm.

**Group**	**Feature**	**Description**	**Calculation**	**Source**
Milk	**hasMilk**	Indication of whether the farm produces commercial milk	*At least one bulk milk analysis recorded in aRes within the year*	aRes
Age & Sex	**pFemaleCalves**	Proportion of animal days of females aged 0–6 months in relation to total animal days of the year	$\frac{FemaleCalvesDays}{TotalAnimalDays}$	AMD
Age & Sex	**pMaleCalves**	Proportion of animal days of males aged 0–6 months in relation to total animal days of the year	$\frac{MaleCalvesDays}{TotalAnimalDays}$	AMD
Age & Sex	**pFemaleYoung**	Proportion of animal days of females aged 7–24 months in relation to total animal days of the year	$\frac{FemaleYoungDays}{TotalAnimalDays}$	AMD
Age & Sex	**pMaleYoung**	Proportion of animal days of males aged 7–24 months in relation to total animal days of the year	$\frac{MaleYoungDays}{TotalAnimalDays}$	AMD
Age & Sex	**pFemaleAdults**	Proportion of animal days of females aged > 24 months in relation to total animal days of the year	$\frac{FemaleAdultsDays}{TotalAnimalDays}$	AMD
Age & Sex	**pMaleAdults**	Proportion of animal days of males aged >24 months in relation to total animal days of the year	$\frac{MaleAdultsDays}{TotalAnimalDays}$	
Cattle Breeds	**pDairyBreed**	Proportion of animal days of dairy breeds in relation to total animal days of the year	$\frac{DairyBreedDays}{TotalAnimalDays}$	AMD
Cattle Breeds	**pBeefBreed**	Proportion of animal days of beef breeds in relation to total animal days of the year	$\frac{BeefBreedDays}{TotalAnimalDays}$	AMD
Cattle Breeds	**pDoubleBreed**	Proportion of animal days of dual-purpose breeds in relation to total animal days of the year	$\frac{DoubleBreedDays}{TotalAnimalDays}$	AMD
Calving	**pCalvedAnimals**	Proportion of animal day of cows which already calved in relation to the total animal days of the year	$\frac{AnimalsCalvedDays}{TotalAnimalDays}$	AMD
Births	**pBirths**	Proportion of births (incl. stillbirths) relative to the annual herd size	$\frac{Births}{TotalHerdSize}$	AMD
Slaughter	**pOutMovesToSLCalves**	Proportion of total number of animals aged 0–6 months leaving the farm directly for slaughter out of total number of slaughtered animals	$\frac{CalvesToSL}{AnimalsToSL}$	AMD
Slaughter	**pOutMovesToSLYoung**	Proportion of total number of animals aged 7–24 months leaving the farm directly for slaughter out of total number of slaughtered animals	$\frac{YoungToSL}{AnimalsToSL}$	AMD
Slaughter	**pOutMovesToSLAdults**	Proportion of total number of slaughtered animals aged >24 months leaving the farm directly for slaughter out of total number of slaughtered animals	$\frac{AdultsToSL}{AnimalsToSL}$	AMD
Animal Movement	**pOutMovesToBirthHerd**	Proportion of animals sold that return to their farm of birth relative to the number of animals leaving the farm in a year	$\frac{AnimalsSoldReturn}{TotalOutMoves}$	AMD
Animal Movement	**inDegree**	Number of farms of origin for arrivals within 1 year	∑*UniqueFarmsArrivals*	AMD
Animal Movement	**outDegree**	Number of farms of origin for departures within 1 year	∑*UniqueFarmsDepartures*	AMD
Animal Movement	**pAnimals10Days**	Proportion of animals sold that were on the farm for 10 days or less relative to the number of animals leaving the farm in a year	$\frac{AnimalsSoldTenDays}{TotalOutMoves}$	AMD
Antibiotics	**pABRearing**	Proportion of antibiotic prescriptions for rearing cattle in relation to the total amount of antibiotic prescriptions per year	$\frac{RearingABPrescriptions}{TotalABPrescriptions}$	IS ABV
Antibiotics	**pABFatteningCalves**	Proportion of antibiotic prescriptions for fattening calves in relation to the total amount of antibiotic prescriptions per year	$\frac{FatteningCalvesABPrescriptions}{TotalABPrescriptions}$	IS ABV
Antibiotics	**pABFatteningYoung**	Proportion of antibiotic prescriptions for cattle fattening in relation to the total amount of antibiotic prescriptions per year	$\frac{FatteningYoungABPrescriptions}{TotalABPrescriptions}$	IS ABV
Antibiotics	**pABMilkCow**	Proportion of antibiotic prescriptions for dairy cows in relation to the total amount of antibiotic prescriptions per year	$\frac{MilkCowABPrescriptions}{TotalABPrescriptions}$	IS ABV
Antibiotics	**pABSuckling**	Proportion of antibiotic prescriptions for suckler cows in relation to the total amount of antibiotic prescriptions per year	$\frac{SucklingABPrescriptions}{TotalABPrescriptions}$	IS ABV

### 2.3 First stage: proof-of-concept

Cattle production types (e.g., calf fattening) were collected and then validated in two stages between October 2022 and November 2023. First, the project was presented to the cVOs in October 2022. Interested members of the cVO provided a list of farms that, according to their knowledge, were considered representative of each of the five specified cattle production types. Within this first stage, we received 227 unique labeled farms from a total of six cVOs. Due to the initial scarcity of farm labels, we used data from these farms in 2020, 2021, and 2022 to train five separate Ranger (21) models using a One-vs.-Rest approach and up-sampling with SMOTE (22). Including data from several years resulted in a total of 632 farm observations, as not every farm had data for 3 years.

As a proof of concept for the cVOs, we applied the models to all eligible cattle farms (*n* = 34,226) in Switzerland. We produced a tagged dataset of all Swiss cattle farms according to the primary five cattle production types. In June 2023 we sent each canton a list of all their farms tagged by our preliminary models, and we asked them to check and validate the tagging for as many cattle farms as possible. The cVOs were satisfied with the preliminary results and sent us a second set of validated farm labels from their jurisdictions for the second stage.

### 2.4 Second stage: final dataset

In the second stage, we received 405 newly validated labeled cattle farms from a total of 14 cVOs. Together with the initial 227 farms, we obtained a total of 632 unique cattle farms that have been labeled and validated by the cVOs. The labels generated from the preliminary models were not used directly for the training. By again utilizing data from 3 years (2020–2022), we identified and excluded 14 farm observations that had extreme values in some features, which could be due to management changes. Some labeled farms had no data in previous years, resulting in an additional 30 missing farm observations. This process resulted in a final dataset comprising 618 unique farms with a total of 1,807 farm observations.

### 2.5 Principal component analysis

To visualize the structure of the labeled production types, we applied Principal Component Analysis (PCA), a dimensionality reduction technique that transforms the original, correlated features into a new set of uncorrelated features called principal components (23). These components are ranked based on the amount of variance they explain, with the first principal component capturing the largest proportion of the variance in the dataset. We used the prcomp function (version 3.6.2) in R to perform PCA on all 23 numerical features in our dataset.

### 2.6 Model selection

We chose three commonly used supervised machine learning models to classify cattle farms. The first was Random Forest (RF), an ensemble learning approach that combines the predictions of many decision trees into a single result (24). The second model was a Support Vector Machine (SVM) algorithm which tries to find the best hyperplane between the production type classes (25). The third model was a neural network, a Multi-layer Perceptron Classifier (MLP), in which each neuron in a layer is connected with a certain weight to each neuron in the next layer (26). We chose these models for their widespread use, versatility, and effectiveness in handling classification tasks in different domains (27).

### 2.7 Model training

The three models were trained using the final dataset of 1,807 farm observations, including the 24 features based on data sourced from AMD, IS ABV, and aRes (Table 2) and the production types provided by the cVO. We split the data into a 70% training set (*n* = 1,262) and a 30% holdout set (*n* = 545). To ensure an equal distribution of farm classes and to ensure that there were no farms with the same AMD number in both sets, we performed a stratified group split. To prevent farms from being grouped within the same fold, the models were trained using a 10-fold cross-validation incorporating group information and optimized for F1-Score using scikit-learn (28). After an initial random search for the hyperparameters we conducted a structured grid search (Supplementary Table 2).

### 2.8 Model evaluation

Models were compared by the following metrics: Accuracy, Precision, Sensitivity, Balanced Accuracy, and F1-Score. These metrics were calculated on the holdout set, while the 95% confidence interval was generated by resampling the training data using bootstrap resampling (*n* = 1,000).

**Accuracy** is the fraction of predictions the model has classified correctly. It is defined as:


$$\begin{array}{l} Accuracy\;=\frac{TP+TN}{TP+TN+FP+FN} \end{array}$$

**Precision** indicates a lower number of falsely values (FP) and is defined as:


$$\begin{array}{l} Precision\;=\frac{TP}{TP+FP} \end{array}$$

**Recall** or **Sensitivity** measures the ability of the model to identify cattle farms and is the true positive rate. A higher recall value indicates a lower number of farms incorrectly tagged as false negatives (FN):


$$\begin{array}{l} Sensitivity\;=\frac{TP}{TP+FN} \end{array}$$

**Specificity** is the true negative rate and measures the proportion of correctly classified negatives. It is defined as:


$$\begin{array}{l} Specificity\;=\frac{TN}{TN+FP} \end{array}$$

**Balanced Accuracy** is the arithmetic mean of the Sensitivity and the Specificity. It is useful when there is an imbalanced distribution of the classes. It is defined as:


$$\begin{array}{l} Balanced\;Accuracy\;=\frac{Sensitivity+Specificity}{2} \end{array}$$

**F1-Score** is the harmonic mean of precision and recall and provides a balanced assessment of a classifier by combining the two metrics:


$$\begin{array}{l} F1\;score=\frac{TP}{TP\;+\;\frac{1}{2}\left(FP\;+\;FN\right)\;} \end{array}$$

### 2.9 Feature importance

To assess the importance of individual features we applied a feature importance framework called SHapley Additive exPlanations (SHAP) (29). SHAP has its foundation in coalitional game theory and provides contribution explanations to analyse the model's output. We used SHAP to quantify the relative importance of features contributing toward a production type label. An average higher (positive) SHAP indicates a higher impact of the feature on the model's production type prediction. SHAP values and plots were produced with the SHAP library in Python (29).

### 2.10 Calibration

Calibration of the models was implemented in scikit-learn (28) using Platt scaling (30) and isotonic regression (31). Furthermore, the Brier Score (32) and Log Loss (33) were calculated, which assessed the agreement between predicted probabilities and actual classification results after calibration. A lower score in both metrics indicates an increased performance of the calibrated classification models. Model calibration is a fundamental practice that ensures the reliability and interpretability of predictive models (34).

## 3 Results

### 3.1 Descriptive summary of reference farms

The final data set included 1,807 observations of 618 farms from 3 years (2020–2022). There were 186 (n-unique = 64) calf fattening farm observations, 772 (n-unique=264) dairy cow farm observations, 224 (n-unique = 76) cattle fattening farm observations, 184 (n-unique = 63) rearing cattle farm observations, and 441(n-unique = 151) suckler cow farm observations. There were 744 farm observations which had at least one commercial milk test within the respective year. Features are described by summary statistics for each production type in Table 3. Notably, in rearing cattle we found the highest median for the proportion of young females (pFemaleYoung) aged 7–24 months with 0.56 (IQR = 0.48–0.62) and the highest median for the proportion of animals that were sold and returned to the farm of birth (pOutMovesToBirthHerd) with 0.44 (IQR = 0.21–0.61). The highest median for the proportion of beef breeds (pBeefBreed) was found for suckler cows with 0.45 (IQR = 0.12–0.66). The highest median for the proportion of young males (pMaleYoung) aged 7–24 months was found in cattle fattening with 0.34 (IQR = 0.03–0.47).

**Table 3:** Summary statistics of all features for each production type in the final dataset (*N* = 1,807 farm observations from 618 individual farms).

**Feature**	**Calf fattening, *n =* 186**	**Dairy cow, *n =* 772**	**Cattle fattening, *n =* 224**	**Rearing cattle, *n =* 184**	**Suckler cow, *n =* 441**
**hasMilk**					
*0*	177 (95%)	57 (7.4%)	215 (96%)	179 (97%)	435 (99%)
*1*	9 (4.8%)	**715** (93%)	9 (4.0%)	5 (2.7%)	6 (1.4%)
**pFemaleCalves**					
*Median (IQR)*	**0.17** (0.12, 0.24)	0.10 (0.08, 0.13)	0.01 (0.0, 0.10)	0.02 (0.00, 0.07)	0.11 (0.08, 0.14)
**pFemaleYoung**					
*Median (IQR)*	0.02 (0.00, 0.18)	0.16 (0.09, 0.22)	0.05 (0.01, 0.33)	**0.56** (0.48, 0.62)	0.13 (0.10, 0.18)
**pFemaleAdults**					
*Mean (IQR)*	0.01 (0.00, 0.33)	**0.48** (0.43, 0.54)	0.00 (0.00, 0.09)	0.13 (0.06, 0.25)	**0.40** (0.36, 0.43)
**pMaleCalves**					
*Median (IQR)*	**0.37** (0.20, 0.55)	0.04 (0.02, 0.07)	0.13 (0.02, 0.32)	0.00 (0.00, 0.00)	0.11 (0.08, 0.14)
**pMaleYoung**					
*Median (IQR)*	0.00 (0.00, 0.02)	0.00 (0.00, 0.02)	**0.34** (0.03, 0.47)	0.00 (0.00, 0.02)	0.09 (0.05, 0.13)
**pMaleAdults**					
*Median (IQR)*	0.00 (0.00, 0.00)	0.00 (0.00, 0.01)	0.00 (0.00, < 0.01)	0.00 (0.00, 0.00)	**0.011** (0.0, 0.02)
**pDairyBreed**					
*Median (IQR)*	0.30 (0.14, 0.49)	**0.48** (0.10, 0.63)	0.09 (0.05, 0.22)	0.40 (0.10, 0.66)	0.00 (0.00, 0.06)
**pBeefBreed**					
*Median (IQR)*	0.11 (0.06, 0.16)	0.00 (0.00, 0.03)	0.19 (0.13, 0.24)	0.00 (0.00, 0.07)	**0.45** (0.12, 0.66)
**pDoubleBreed**					
*Median (IQR)*	0.13 (0.08, 0.26)	0.13 (0.03, 0.54)	0.12 (0.07, 0.17)	0.13 (0.01, 0.40)	0.03 (0.00, 0.11)
**pCalvedAnimals**					
*Median (IQR)*	0.00 (0.00, 0.19)	**0.39** (0.36, 0.43)	0.00 (0.00, 0.06)	0.02 (0.00, 0.18)	**0.32** (0.29, 0.35)
**pBirths**					
*Median (IQR)*	0.00 (0.00, 0.11)	**0.28** (0.24, 0.31)	0.00 (0.00, 0.00)	0.00 (0.00, 0.00)	**0.25** (0.21, 0.28)
**pOutMovesToSLCalves**					
*Median (IQR)*	**0.65** (0.58, 0.68)	0.00 (0.00, 0.29)	0.00 (0.00, 0.00)	0.00 (0.00, 0.00)	0.00 (0.00, 0.06)
**pOutMovesToSLYoung**					
*Median (IQR)*	0.04 (0.01, 0.14)	0.00 (0.00, 0.22)	**0.69** (0.62, 0.69)	0.00 (0.00, 0.57)	**0.51** (0.18, 0.61)
**pOutMovesToSLAdults**					
*Median (IQR)*	0.00 (0.00, 0.02)	**0.48** (0.23, 0.69)	0.00 (0.00, 0.07)	0.00 (0.00, 0.06)	0.17 (0.08, 0.38)
**pOutMovesToBirthHerd**					
*Median (IQR)*	0.00 (0.00, 0.00)	0.00 (0.00, 0.02)	0.00 (0.00, 0.00)	**0.44** (0.21, 0.61)	0.00 (0.00, 0.02)
**inDegree**					
*Median (IQR)*	**4.76** (2.50, 5.87)	1.61 (1.10, 2.20)	3.28 (2.30, 4.41)	1.61 (1.10, 2.20)	1.39 (1.10, 1.95)
**outDegree**					
*Median (IQR)*	1.10 (0.00, 1.95)	**2.56** (1.95, 3.04)	0.69 (0.00, 1.61)	1.61 (1.10, 2.08)	1.39 (0.69, 1.79)
**pAnimals10Days**					
*Median (IQR)*	0.00 (0.00, 0.01)	0.00 (0.00, 0.04)	0.00 (0.00, 0.00)	0.00 (0.00, 0.07)	0.00 (0.00, 0.04)
**pABRearing**					
*Median (IQR)*	0.00 (0.00, 0.00)	0.01 (0.00, 0.05)	0.00 (0.00, 0.00)	0.00 (0.00, 0.02)	0.00 (0.00, 0.00)
**pABFatteningCalves**					
*Median (IQR)*	**0.42** (0.00, 1.07)	0.00 (0.00, 0.01)	0.01 (0.00, 0.21)	0.00 (0.00, 0.00)	0.00 (0.00, 0.00)
**pABFatteningYoung**					
*Median (IQR)*	0.00 (0.00, 0.00)	0.000 (0.00, 0.00)	0.00 (0.00, 0.01)	0.00 (0.00, 0.00)	0.00 (0.00, 0.00)
**pABMilkCow**					
*Median (IQR)*	0.00 (0.00, 0.03)	**0.14** (0.04, 0.24)	0.00 (0.00, 0.00)	0.00 (0.00, 0.00)	0.00 (0.00, 0.01)
**pABSuckling**					
*Median (IQR)*	0.00 (0.00, 0.00)	0.00 (0.00, 0.00)	0.00 (0.00, 0.00)	0.00 (0.00, 0.00)	**0.02** (0.00, 0.09)

Bold values represent the largest or most interesting value in each row.

### 3.2 Clustering patterns in production types

**Figure 1:**
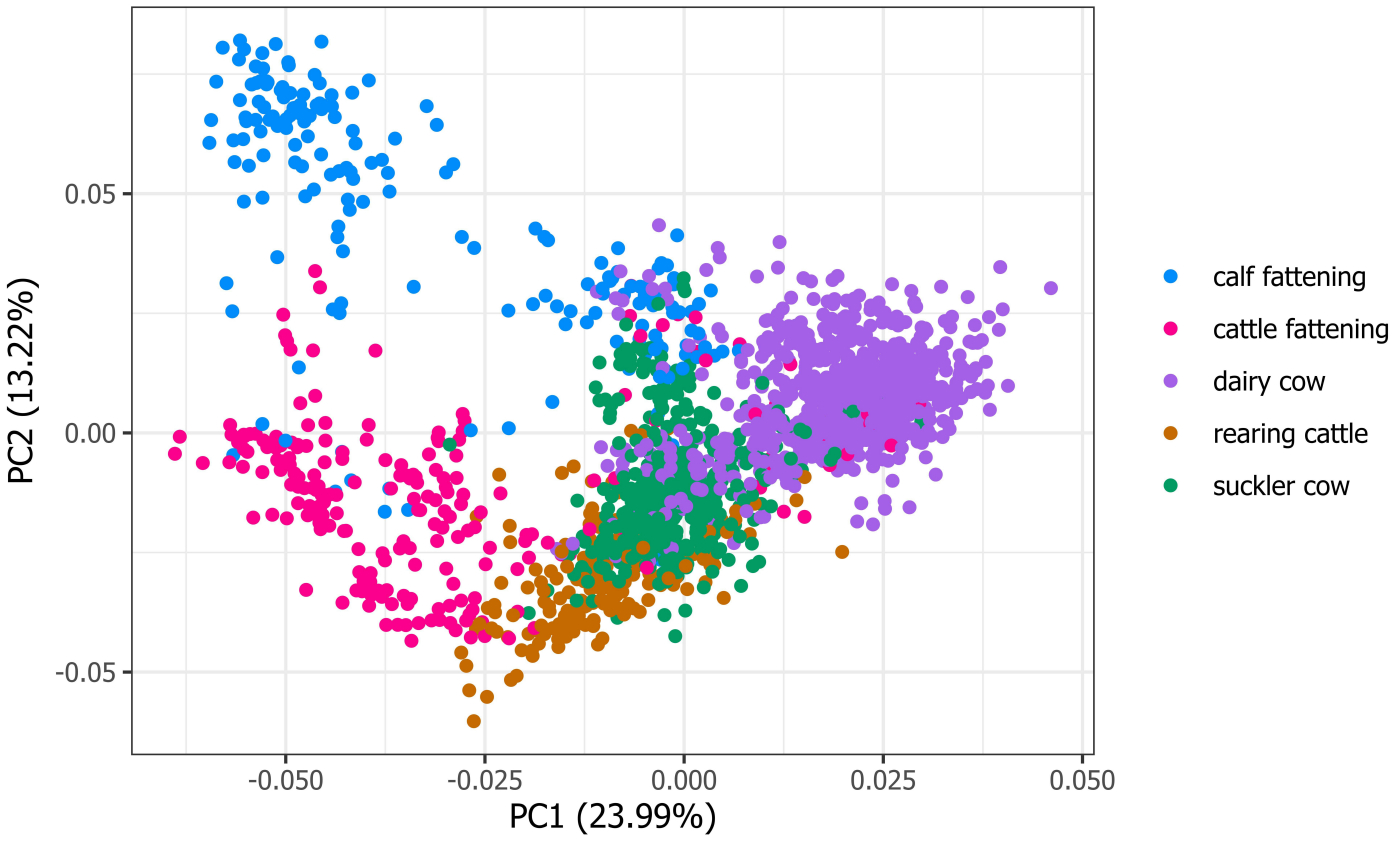
Principal Component Analysis (PCA) applied to the normalized features illustrate the relationships between production types, with each dot representing a farm within a specific year (*N* = 1,807). The dots are color-coded to represent the corresponding cattle production types as labeled by the cantonal Veterinary Offices (cVO), offering an overview of the data structure.

The PCA plot, based on all numerical features and farm observations, showed distinct clusters, though some overlap existed among production types (Figure 1). Notably, the first two principal components only accounted for 37.21% of the total variance. The primary contributors to PC1 were the proportion of female adults (pFemaleAdults = 0.382) and the proportion of calved animals (pCalvedAnimals = 0.369), while the proportion of animals leaving the farm for slaughter (pOutMovesToSLCalves=0.424) and the proportion of antibiotic prescriptions for fattening calves (pABFatteningCalves = 0.347) contributed most (0.347) to PC2 (Supplementary Table 3).

### 3.3 Model evaluation

The ability of the models to classify farms was evaluated on 30% of the unseen data, which serves as a proxy for the other unlabelled cattle farms in Switzerland. Random Forest achieved the highest accuracy of 0.914 (95% CI: 0.890, 0.938) and balanced accuracy of 0.882 (95% CI: 0.844, 0.917) (Table 4). The lowest accuracy of 0.883 (95% CI: 0.855, 0.910) was obtained by the neural network MLP. SVM with 0.851 (95% CI: 0.808, 0.889) and MLP with 0.853 (95% CI: 0.814, 0.890) had similar balanced accuracies. The highest precision (0.880, 95% CI: 0.843, 0.915) and sensitivity (0.882, 95% CI: 0.844, 0.917) was accomplished by Random Forest. This is also reflected by Random Forest reaching the highest F1-Score of 0.879 (95% CI: 0.841, 0.913). While the performance of SVM was not exceptional, it still achieved a respectable F1-Score of 0.835 (95% CI: 0.793, 0.874).

**Table 4:** Model evaluation scores of all three classifiers.

**Model**	**Accuracy (95% CI)**	**Balanced accuracy (95% CI)**	**Precision (95% CI)**	**Sensitivity (95% CI)**	**F1-Score (95% CI)**
Random forest	0.914 (0.890, 0.938)	0.882 (0.844, 0.917)	0.880 (0.843, 0.915)	0.882 (0.844, 0.917)	0.879 (0.841, 0.913)
Support vector machine	0.888 (0.859, 0.914)	0.851 (0.808, 0.889)	0.825 (0.788, 0.867)	0.851 (0.808, 0.889)	0.835 (0.793, 0.874)
Neural network	0.883 (0.855, 0.910)	0.853 (0.814, 0.890)	0.828 (0.790, 0.868)	0.853 (0.814, 0.890)	0.839 (0.801, 0.876)

The 95% confidence intervals are shown in parentheses.

**Figure 2:**
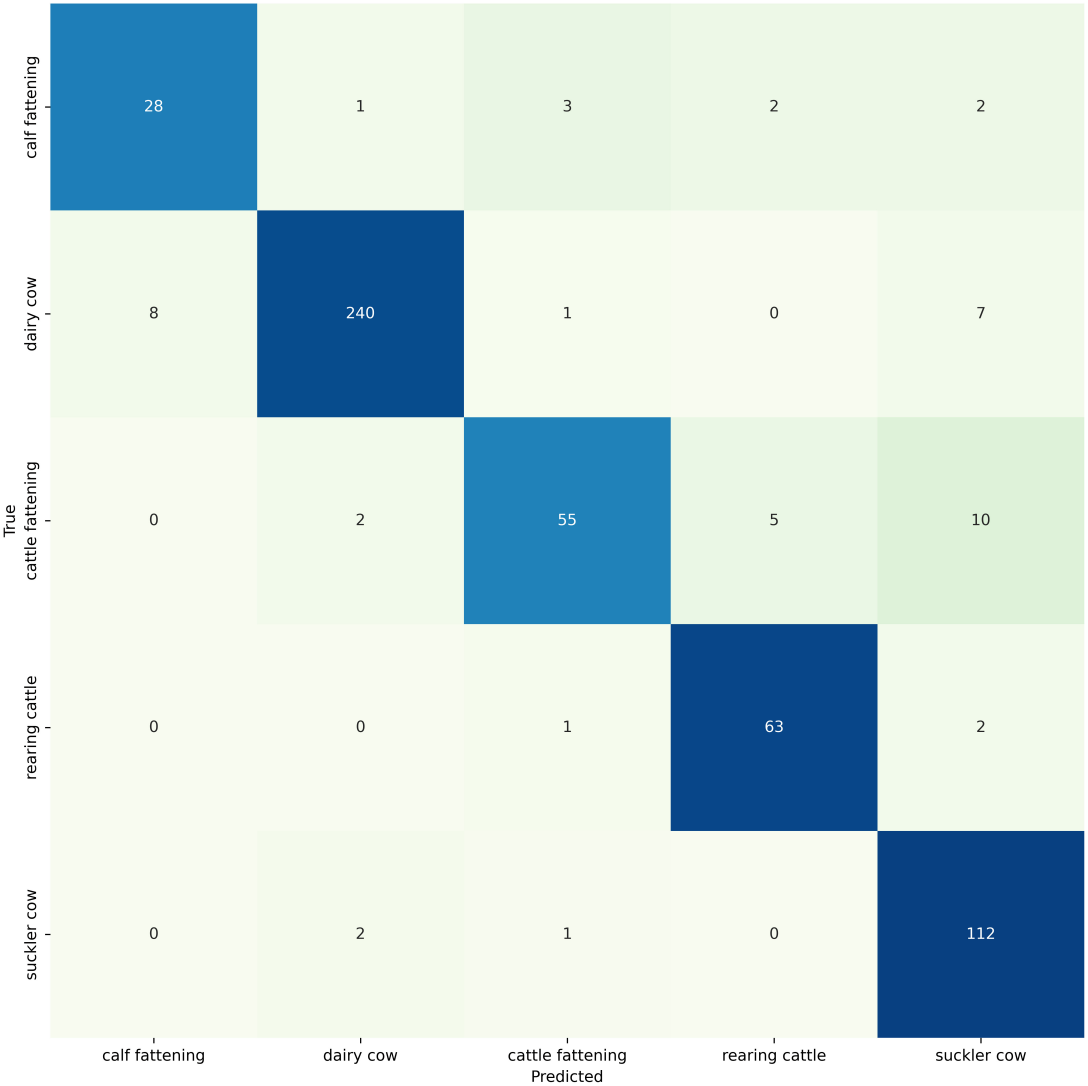
Confusion Matrix showing the performance of the Random Forest classifier on the holdout set (*n* = 545). The y-axis corresponds to the true production types defined by the cantons, while the x-axis represents the predicted production types by the Random Forest.

Random Forest could correctly identify 240 out of the 256 dairy farm observations, in the holdout set (Figure 2). The absolute largest error was made in calf fattening, cattle fattening and suckler cow farm observations. Random Forest misclassified 8 dairy cow farms observations as calf fattening and 7 dairy cow farm observations as suckler cow farms. Furthermore, it misclassified 10 cattle fattening farm observations as suckler cow farms. Specifically, Random Forest achieved a label specific accuracy of 0.78 for calf fattening, 0.94 for dairy cow, 0.76 for large fattening cattle, 0.96 for rearing cattle and 0.97 for suckler cow.

### 3.4 Feature importance

**Figure 3:**
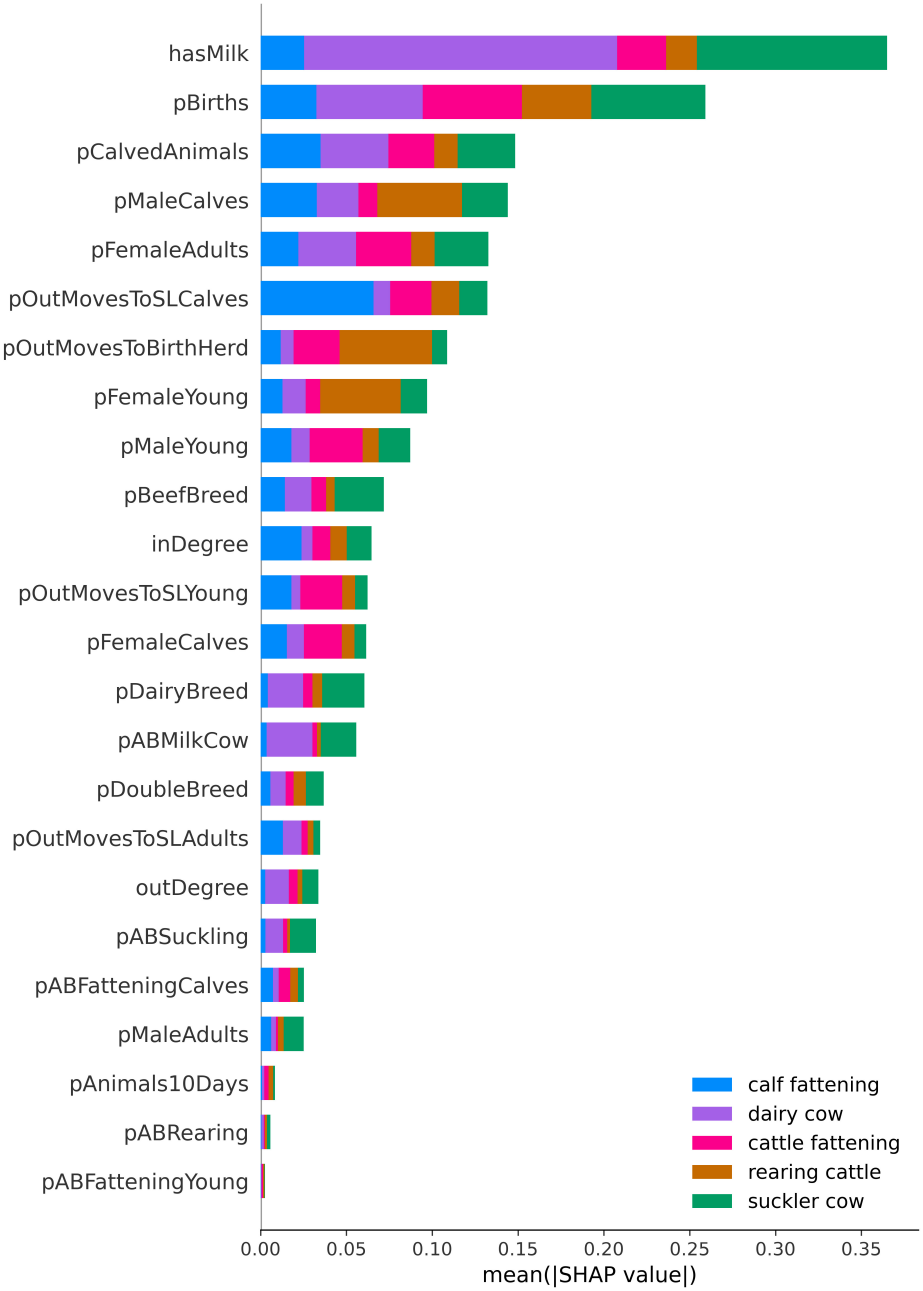
SHapley Additive Explanations (SHAP) value of the Random Forest classifier. The bar plot displays feature importance, calculated as the sum of the mean absolute SHAP-values for each feature across all production types (*n* = 5). Color indicates the mean absolute SHAP value per production type. Features are ranked according to the sum of the mean absolute SHAP values for all observations and production types.

The mean absolute SHAP value quantifies the typical expected impact of each feature on the outcome of the model (Figure 3). Overall, whether commercial milk was produced (hasMilk) and the proportion of births (pBirths) were the most influential features for both dairy (0.18) and suckler cow (0.11) farms (Supplementary Table 4). For calf fattening, the proportion of animals leaving the farm for slaughter (pOutMovesToSLCalves) was the most impactful (0.07) feature. Proportion of births (pBirths = 0.06) and proportion of young males (pMaleYoung = 0.03) aged 7–24 months were the most impactful features for cattle fattening. The proportion of animals that were sold and returned to the farm of birth (pOutMovesToBirthHerd = 0.05) and the proportion of males aged 0–6 months (pMaleCalves = 0.05) were the most influential features for rearing cattle.

### 3.5 Calibration

Table 5 shows that applying calibration with sigmoid regression slightly improved Random Forest' log loss to 0.353 (−0.012). Sigmoid regression increased the precision of Random Forest to 0.881 (+0.002) and the F1-Score to 0.878 (+0.001). Isotonic also improved the log loss to 0.361 (−0.004) but had a decrease in precision to 0.865 (−0.014).

**Table 5:** Evaluation before and after calibration of the Random Forest (RF) classifier.

**Random Forest**	**Log Loss**	**Accuracy**	**Precision**	**Recall**	**F1-Score**
Uncalibrated	0.365	0.912	0.879	0.881	0.877
Isotonic	0.361	0.901	0.865	0.867	0.864
Sigmoid	0.353	0.912	0.881	0.881	0.878

### 3.6 Classification output

One aim of this study was to provide each cVO with a simple overview of the probabilities of for each production type. The classification output is a list of all the farms in their canton with all the production type probabilities (Table 6). It is also useful to see if the decision was not as clear and there is a possibility of a mixed production, as shown in Table 6 with Farm ID 4.

**Table 6:** Example of the final classification output for five anonymised farms, illustrating practical results that can be used in decision making.

**Farm ID**	**Production type probability**					**Production type prediction**
	**Calf fattening**	**Dairy cow**	**Cattle fattening**	**Rearing cattle**	**Suckler cow**	
1	0.00	**0.91**	0.06	0.00	0.03	Dairy cow
2	**0.45**	0.19	0.14	0.03	0.19	Calf fattening
3	0.00	0.09	0.01	0.02	**0.88**	Suckler cow
4	0.10	0.37	0.12	**0.39**	0.02	Rearing cattle
5	0.05	0.19	**0.70**	0.05	0.01	Cattle fattening

Values in bold represent the highest probability and the corresponding prediction for each production type.

## 4 Discussion

Using expert knowledge, we were able to outline five cattle production types which are most relevant for activities of veterinary services in Switzerland. With three national datasets under public law and therefore available for administrative purposes, we trained a Random Forest algorithm that reliably classified these five types. This improves the classification of cattle production types in Switzerland greatly compared to the two production types (milk/non-milk) available so far. After working with the cVOs and using their expert knowledge of farms within their jurisdiction, we had labeled production types for 618 unique farms which allowed for well-trained models and proved to be a promising tool for classifying cattle production types.

The summary statistics (Table 3) of the features were consistent with the expected production type characteristics according to expert knowledge (Table 1). Many features were found to be distinctive in their values for one or two production types. The commercial milk tag (hasMilk) was mainly found on dairy farms (93%). The proportion of young females (pFemaleYoung) had the highest median in rearing cattle and the proportion of adult females (pFemaleAdults) had the highest occurrence on dairy cow farms, which corresponds to the expected sex distribution by experts. The highest median for the proportion of male calves (pMaleCalves) with 0.37 and a mixed distribution for beef and dairy breed were observed on calf fattening farms. The proportion of young males (pMaleYoung) aged 7–24 months was highest on cattle fattening farms, for the proportion of adult males (pMaleAdults), we observed the highest median value (0.011) on suckler cow farms, which is probably due to the presence of at least one adult bull on these types of farms and less artificial insemination compared to dairy farms. The proportion of young animals leaving the farm for slaughter (pOutMovesToSLCalves) had its highest value on calf fattening farms which is also concurrent with experts' opinion. For the proportion of young animals (7–24 months) leaving the farm for slaughter (pOutMovesToSLYoung) we found a median of 0.69 for cattle fattening farms, which is also expected given the output of such farms to slaughterhouses. The two highest values for the feature representing the arrivals on a farm (inDegree) were found on calf fattening farms and cattle fattening farms, which conforms to the expert opinion that these types of farms acquire the most additional cattle from different farms.

We observed the same patterns in our SHAP feature contribution analysis, where the proportion of young animals (7–24 months) leaving the farm for slaughter (pOutMovesToSLYoung) was the most influential feature in classifying cattle fattening farms. Interestingly, for rearing farms, the proportion of male calves (pMaleCalves) was one of the most contributing features with a median of 0.00, meaning that a low value helped to differentiate them from the other classes. While the feature for commercial milk (hasMilk) doesn't surprise with its high influence to distinguish dairy farms, we can say that the proportion of births (pBirths) is the second most influential feature in our data set; either to characterize rearing farms with a lack of births or due to the higher pBirths in suckler cows. We also found the same trends as described by the experts when we examined the direction of SHAP values for each feature and class, and their association with high or low data points (Supplementary Figures 1–5). Overall, this means that the subset we obtained from the cVOs is representative of the topology of Swiss cattle farms and that our algorithm classifies each production type with the biologically most meaningful features.

The Random Forest algorithm reliably classified the five production types with an overall accuracy of 0.914 (95% CI: 0.890, 0.938) and a F1-Score of 0.879 (95% CI: 0.841, 0.913). In comparison to the other models, it had not only a higher accuracy but also a higher F1-Score. Although we could slightly improve the performance in most metrics by calibrating the Random Forest classifier with an isotonic regression, the difference is not remarkable.

The extended production type classification will help the FSVO design more appropriate surveillance programmes considering known risk factors for disease introduction and spread for the different production types. It also helps the cVOs to specifically target certain types of farms for risk-based food safety and animal welfare inspections. Our classification should ultimately lead to better allocation of resources, improved disease surveillance and control, and overall improvements in animal health management. For example, the identification of farms by type could facilitate more effective epidemiological investigations when positive results for Bovine Viral Diarrhea (BVD) or other infectious diseases such as Infectious Bovine Rhinotracheitis (IBR) and Enzootic Bovine Leukosis (EBL) are detected. In the future, farms could be classified as pure fattening and then excluded from certain eradication programmes (e.g., BVD eradication) due to their limited role in disease transmission, which could lead to cost savings. With the current broad classification of production types, it is difficult to make an objective and fair comparison of antibiotic use on farms, as the production type plays a major role in the frequency and type of antibiotic prescriptions.

The Random Forest algorithm can easily be applied to data from a new year. This enables the FSVO to continuously categorize newly registered farms, track farms that have changed their production type over time and monitor shifts in the prevalence of different production types on Swiss cattle farms. Instead of relying on farmers' self-declaration, the FSVO can use a tool that classifies farms independently of survey participation and compliance as most features used in the algorithm are collected from the movement database, the slaughterhouse or milk collection centers and do not rely on additional self-declaration. This could ensure a more objective and consistent classification of farms, reducing the subjectivity and potential bias associated with self-declaration. However, as the demographics of the herds may change, a constant feedback loop with the cVOs should be established to allow for the contentious collection of new labels as well as the constant validation of labels over time.

One of the drawbacks is that we have received well-known farms (to the cVO) that are probably well structured, whereas the rest of the Swiss cattle sector may be more diverse. However, the currently used categorization (milk or non-milk) puts all non-dairy farm in one broad category—any additional information is useful. Furthermore, due to their specific nature, the models presented here, their results and expert opinions cannot be directly generalized beyond Switzerland. However, other countries or regions may adopt similar strategies in collaboration with their veterinary authorities. In this context, this study provides a flexible framework, together with a set of key features and their relative importance for classification, providing a valuable guide for future implementations.

Due to the limited sample size of labeled farms (*n* = 618), we used data spanning 3 years. While this approach may potentially overestimate some effects, it also addresses the variability of farm characteristics and shifting herd demographics over time, providing a more comprehensive view of farm variation. To address potential bias related to production type, we used group k-fold cross-validation during the training phase. This method ensures that data from the same farm does not appear in both the training and validation folds. In addition, we ensured that the initial training-test split was done in such a way that all data points from the same farm remained within the same group.

The limited sample size of the labeled farms could also be unfavorable for generalization to the whole of Switzerland, but we believe that with 14 cVOs providing us with known representative samples in their jurisdiction, we have a broad and characteristic sample. It should also be noted that the limited number of experts available to characterize production types (*n* = 4) may affect the classification accuracy and generalisability within the topology of Swiss farms. While the labels were designed by only four experts, the actual labeling was done by the cVOs, which we believe strengthens the process by distributing the labeling to more people. Further biases may have been introduced by the fact that the proof-of-concept labels, produced and sent in the first stage, were generated by Ranger models, which are themselves Random Forest algorithms. On the one hand, the sent labels may have influenced the validation in the second stage by cVOs, and on the other hand, due to their similarity to Ranger, they may have favored the Random Forest algorithm with the final dataset compared to the others. However, we believe that these biases are negligible, as the labels in the final dataset were manually validated by the cVOs and none of the labels generated in the first stage were directly used in the second stage.

The main difference with Brock et al. (18) is that we utilize a supervised approach and have an a priori number of production types. Our approach lacks the exploratory nature of the self-organizing maps (SOMs), where experts can help decision making with the very practical graphical representation of the SOMs. Although their approach is more nuanced, we believe that our method with fewer initial categories is a pragmatic approach which allows direct application of the results with an immediate improvement compared to the current classification. If farms are classified into data-based categories with no direct interpretation, the categories are much less likely to be used by veterinary authorities in their daily work.

In the current version, the classification algorithm is only trained to categorize farms into five pre-defined primary production types. However, mixed production types with several animal categories exist, such as dairy farms with calf fattening. As shown in Figure 1, while there are clear clusters, there is also some overlap of blue dots (representing calf fattening) which have formed an intermediate cluster between them and the dairy farm cluster (purple). For many epidemiological purposes, identifying the primary production type is sufficient. However, sometimes it may also be important to identify farms with mixed production types. To fully understand all the activities of a farm, it would be necessary to take the secondary production type into account. In this study, and in the output for the cVOs, we included the model-estimated probabilities for each of the production types. This allows the potential identification of mixed farms, defined as those with similar probability values for two production types. However, to improve the accuracy of the secondary type classification, we think that an additional label clearly identifying the secondary type would be required and further research would be needed to test the accuracy of this extended classification.

Furthermore, additional features could be explored to improve the performance of the classification. Most of the antibiotic use features did not contribute much to the overall classification (Supplementary Table 4), but more nuanced features could be extracted from IS ABV, such as specific antibiotic treatments that would be more common in certain production types. It's important to note that the variables reflect the proportion of prescriptions for a particular production type on a farm. For example, a suckler cow farm that did not use antibiotics is still classified as such, even if it had a low value in the variable representing antibiotic use in suckler cows (pABSuckling). Antibiotic treatments are more common on dairy farms and the variable representing them (pABMilkCow), when low, added to the classification effect (Supplementary Figure 5), helping to distinguish suckler from dairy farms. Due to the nature of antibiotic treatments, we believe that these variables are more of an auxiliary measure, making it easier to differentiate farms that have used antibiotics.

## 5 Conclusion

In this study, we were able to use expert knowledge to specify the five primary production types of cattle farms in Switzerland. The machine learning model presented here can reliably classify production types using three national datasets. This represents a considerable improvement compared to the two types that could be defined so far. This improved classification of production types will enhance the surveillance of cattle diseases and facilitate the work of the cVO in epidemiological investigations by integrating it into existing surveillance systems. Further efforts are needed for an even better and more up-to-date classification of production types. The model could be adapted to other species and for the classification into other production categories. The results cannot be directly transferred to other countries, because the availability of data and the relevant production types differ across countries. However, the methodology is flexible and can easily be adapted to other datasets.

## Data Availability

The datasets presented in this article are not readily available because they contain personal data of farms, farmers, and veterinarians. However, requests to access the datasets & code can be directed to the appropriate channels of the FSVO. Requests to access the datasets should be directed to data@admin.blv.ch.
