# Supplementary material for: Improved cattle farm classification: leveraging machine learning and linked national datasets
Source: Front Vet Sci. 2025 Feb 5;12:1517173. doi: 10.3389/fvets.2025.1517173 (PMC11836822; doi:10.3389/fvets.2025.1517173)
Supplement: Supplementary file 1 [file Table_1.docx]

Supplementary Material

# Supplementary Tables

Supplementary Table 1: Breeds and their type of use category.

| **Breed** | **Type of use** |
| --- | --- |
| Abondance | dual purpose |
| Angler | dual purpose |
| Angus | beef |
| Aubrac | beef |
| Ayrshire | dairy |
| Bazadaise | beef |
| Bison | beef |
| Blonde d'Aquitaine | beef |
| Bordelaise | dual purpose |
| Braunvieh | dual purpose |
| Charolais | beef |
| Chianina | beef |
| Crossbreed | unknown |
| Daenische Rotbunte | dual purpose |
| Dahomey | beef |
| Dexter | beef |
| Eringer | beef |
| Evolene | beef |
| Galloway | beef |
| Gasconne | beef |
| Gelbvieh | beef |
| Grauvieh | beef |
| Guernsey | dairy |
| Hereford | beef |
| Highland Cattle | beef |
| Hinterwaelder | beef |
| Holstein | dairy |
| INRA95 | beef |
| Jersey | dairy |
| Kiwicross | dual purpose |
| Limousin | beef |
| LowLine | beef |
| Luing | beef |
| Maine Anjou | beef |
| Marchigiana | beef |
| Montbeliard | dairy |
| Murnau Werdenfelser | dairy |
| Normande | dairy |
| Norwegische Rotbunte | dairy |
| Original Braunvieh | dairy |
| Other | unknown |
| Parthenaise | beef |
| Piemontese | beef |
| Pinzgauer | beef |
| Pustertaler Sprinzen | beef |
| Red Holstein | dairy |
| Romagnola | beef |
| Rotes Hoehenvieh | beef |
| Rotfleckvieh | dual purpose |
| Salers | beef |
| Schwedische Rotbunte | dual purpose |
| Shorthorn | beef |
| Simmental | dual purpose |
| Swiss Fleckvieh | dairy |
| Tarentaise | dairy |
| Texas Longhorn | beef |
| Tuxer | beef |
| Undefined | unknown |
| Valdostana | dual purpose |
| Vosgienne | beef |
| Wagyu | beef |
| Wasserbueffel | dairy |
| Weissblaue Belgier | beef |
| Welsh Black | beef |
| Yak | dual purpose |
| Zebu | dual purpose |
| Zwergzebu | dual purpose |

Supplementary Table 2: Selected parameters after grid search for all models.

| **Model** | **Hyperparameters** | **Explored hyperparameter values** | **Selected hyperparameters** |
| --- | --- | --- | --- |
| RF | n_estimators | [100, 150, 200, 250, 300, 350, 400, 450] | 300 |
|  | max_depth | [None, 5, 10, 15, 20, 25, 30, 40, 50, 60, 70, 80, 90, 100] | 30 |
|  | max_features | ['sqrt'] | sqrt |
|  | min_samples_split | [1, 2, 3, 4, 5] | 4 |
|  | class_weight | ['balanced', None] | balanced |
| SVM | kernel | ['linear', 'rbf'] | linear |
|  | C | [15, 20, 25, 30, 35, 40] | 25 |
|  | degree | [1,2, 3, 4] | 1 |
|  | gamma | ['auto', 0.1, 0.5, 0.6] | auto |
|  | coef0 | [0.0, 0.1, 0.5] | 0 |
| MLP | max_iter | [1000] | 1000 |
|  | hidden_layer_sizes | [(50,), (100,), (50,20,5,)] | (100,) |
|  | activation | ['tanh', 'relu'] | tanh |
|  | solver | ['sgd', 'adam'] | adam |
|  | alpha | [0.0001, 0.001, 0.01] | 0.01 |
|  | n_iter_no_change | [500] | 500 |

Supplementary Table 3: PCA loadings table of the two leading components. Loadings indicate how much each variable contributes to a particular principal component (PC).

| **Feature** | **PC1** | **PC2** |
| --- | --- | --- |
| pBirths | 0.32586 | 0.074367 |
| pCalvedAnimals | 0.369248 | 0.031918 |
| pMaleCalves | -0.2885 | 0.332314 |
| pFemaleAdults | 0.382069 | 0.052056 |
| pOutMovesToSLCalves | -0.0686 | 0.423741 |
| pOutMovesToBirthHerd | -0.03434 | -0.24273 |
| pFemaleYoung | -0.02755 | -0.30252 |
| pMaleYoung | -0.23618 | -0.20127 |
| pBeefBreed | -0.12521 | -0.16448 |
| inDegree | -0.26835 | 0.301952 |
| pOutMovesToSLYoung | -0.21133 | -0.26292 |
| pFemaleCalves | -0.01943 | 0.33841 |
| pDairyBreed | 0.10802 | 0.128402 |
| pABMilkCow | 0.235096 | 0.157727 |
| pDoubleBreed | 0.104699 | 0.076557 |
| pOutMovesToSLAdults | 0.297179 | 0.012212 |
| outDegree | 0.272666 | 0.116492 |
| pABSuckling | -0.01132 | -0.06321 |
| pABFatteningCalves | -0.24344 | 0.346678 |
| pMaleAdults | 0.006208 | -0.12676 |
| pAnimals10Days | 0.064505 | -0.00637 |
| pABRearing | 0.109415 | 0.080399 |
| pABFatteningYoung | -0.13032 | 0.015361 |

Supplementary Table 4: Mean absolute SHAP values. Each row corresponds to a feature, with the mean absolute SHAP value indicating its importance.

| **Features** | **Calf fattening** | **Dairy cow** | **Cattle fattening** | **Rearing cattle** | **Suckler cow** |
| --- | --- | --- | --- | --- | --- |
| hasMilk | 0.02541 | 0.18235 | 0.02854 | 0.01790 | 0.11083 |
| pBirths | 0.03252 | 0.06193 | 0.05780 | 0.04048 | 0.06644 |
| pCalvedAnimals | 0.03497 | 0.03947 | 0.02677 | 0.01363 | 0.03352 |
| pMaleCalves | 0.03275 | 0.02423 | 0.01098 | 0.04937 | 0.02674 |
| pFemaleAdults | 0.02196 | 0.03369 | 0.03208 | 0.01368 | 0.03134 |
| pOutMovesToSLCalves | 0.06575 | 0.00972 | 0.02402 | 0.01626 | 0.01639 |
| pOutMovesToBirthHerd | 0.01173 | 0.00752 | 0.02681 | 0.05389 | 0.00870 |
| pFemaleYoung | 0.01274 | 0.01349 | 0.00846 | 0.04698 | 0.01532 |
| pMaleYoung | 0.01798 | 0.01063 | 0.03078 | 0.00942 | 0.01834 |
| pBeefBreed | 0.01415 | 0.01553 | 0.00853 | 0.00488 | 0.02869 |
| inDegree | 0.02381 | 0.00644 | 0.01034 | 0.00961 | 0.01441 |
| pOutMovesToSLYoung | 0.01791 | 0.00523 | 0.02446 | 0.00741 | 0.00738 |
| pFemaleCalves | 0.01536 | 0.00993 | 0.02196 | 0.00742 | 0.00684 |
| pDairyBreed | 0.00418 | 0.02058 | 0.00543 | 0.00573 | 0.02455 |
| pABMilkCow | 0.00348 | 0.02678 | 0.00259 | 0.00227 | 0.02068 |
| pDoubleBreed | 0.00570 | 0.00894 | 0.00465 | 0.00710 | 0.01047 |
| pOutMovesToSLAdults | 0.01301 | 0.01085 | 0.00358 | 0.00331 | 0.00399 |
| outDegree | 0.00272 | 0.01380 | 0.00498 | 0.00277 | 0.00940 |
| pABSuckling | 0.00283 | 0.01030 | 0.00246 | 0.00159 | 0.01510 |
| pABFatteningCalves | 0.00725 | 0.00329 | 0.00677 | 0.00446 | 0.00345 |
| pMaleAdults | 0.00621 | 0.00271 | 0.00124 | 0.00336 | 0.01156 |
| pAnimals10Days | 0.00090 | 0.00126 | 0.00247 | 0.00272 | 0.00097 |
| pABRearing | 0.00056 | 0.00133 | 0.00063 | 0.00118 | 0.00197 |
| pABFatteningYoung | 0.00037 | 0.00014 | 0.00098 | 0.00073 | 0.00033 |

## Supplementary Figures


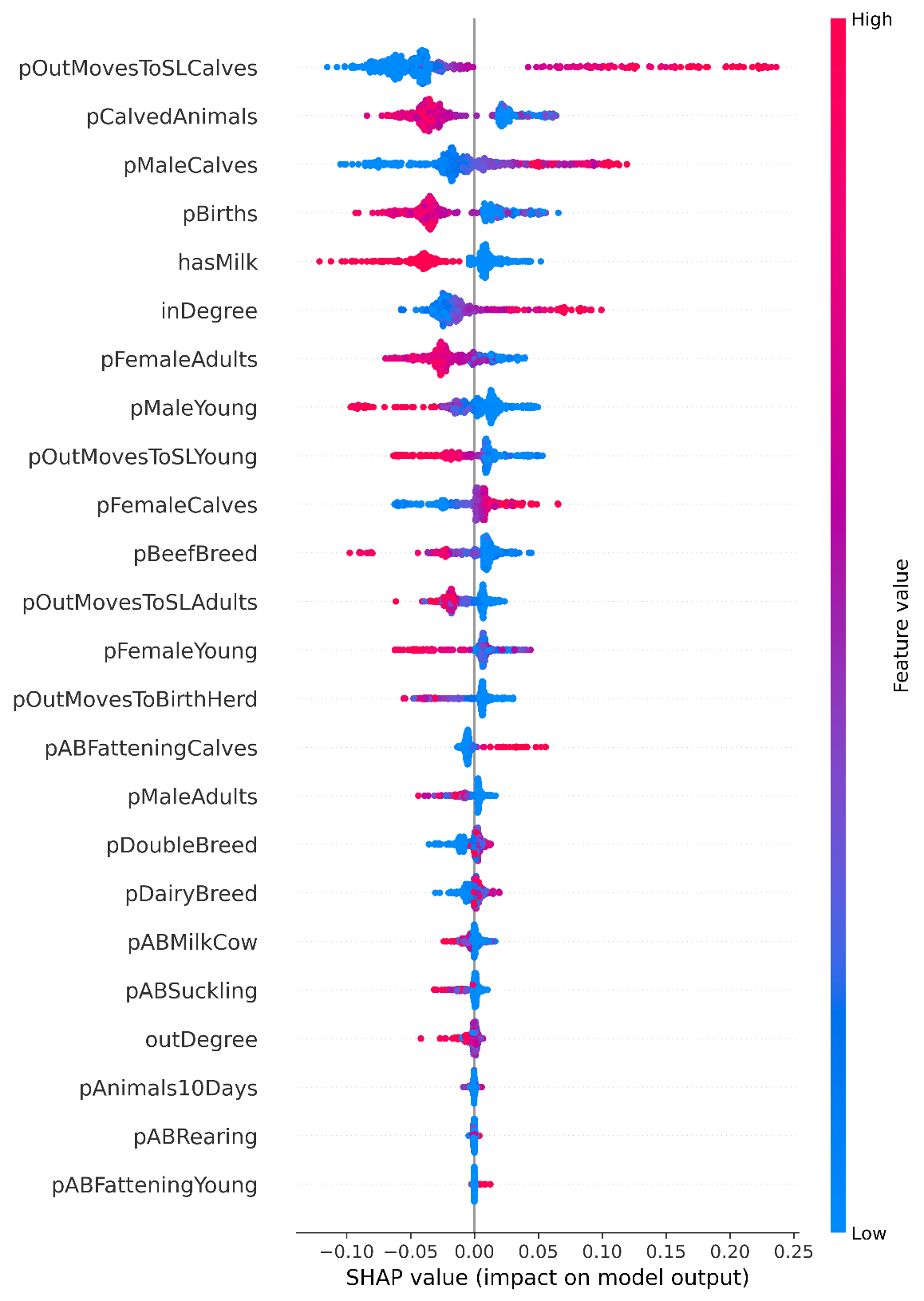


Supplementary Figure 1: Beeswarm plot of SHAP values by feature, illustrating their direction and impact on the prediction of the calf fattening class.


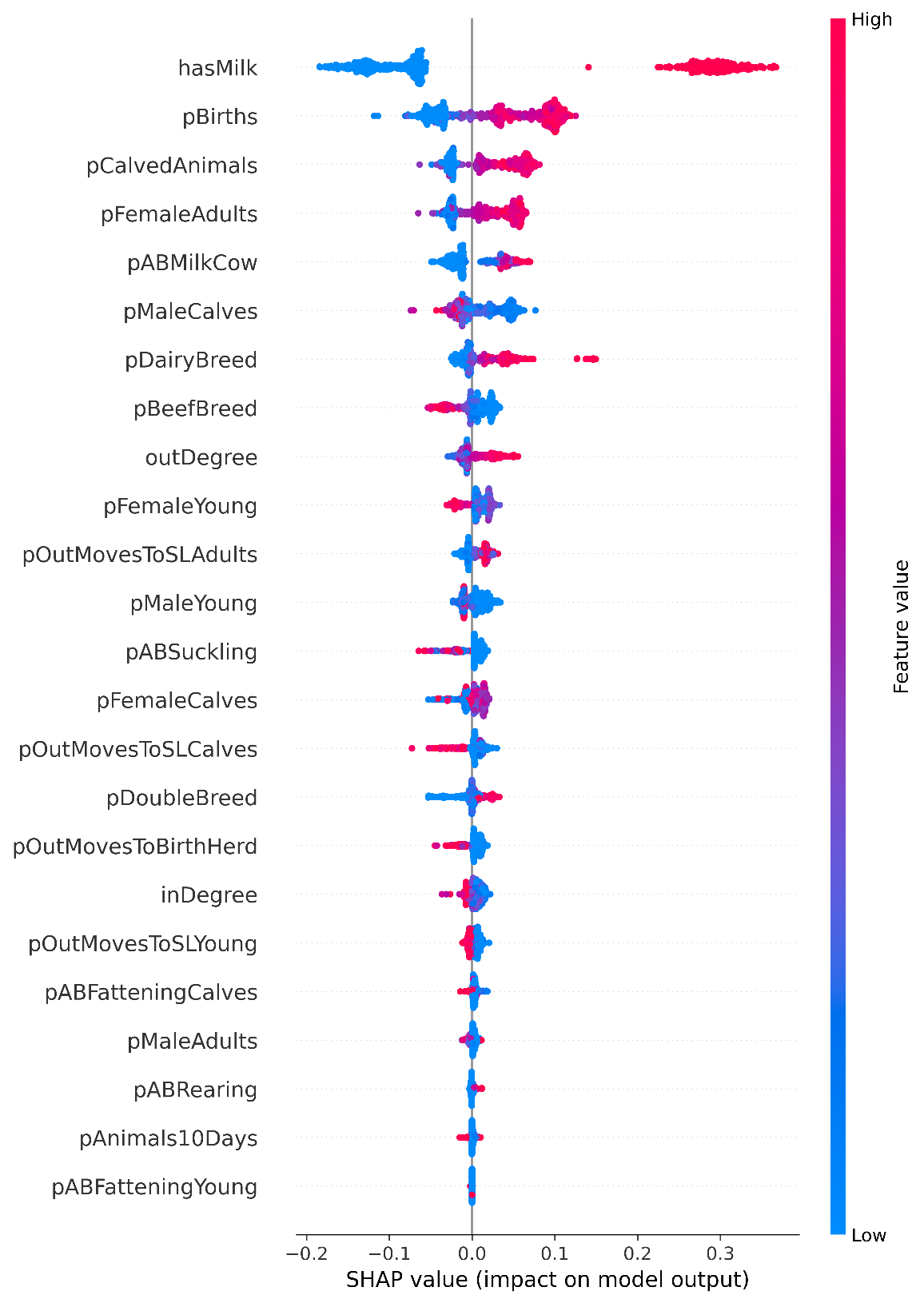


Supplementary Figure 2: Beeswarm plot of SHAP values by feature, illustrating their direction and impact on the prediction of the dairy cow class.


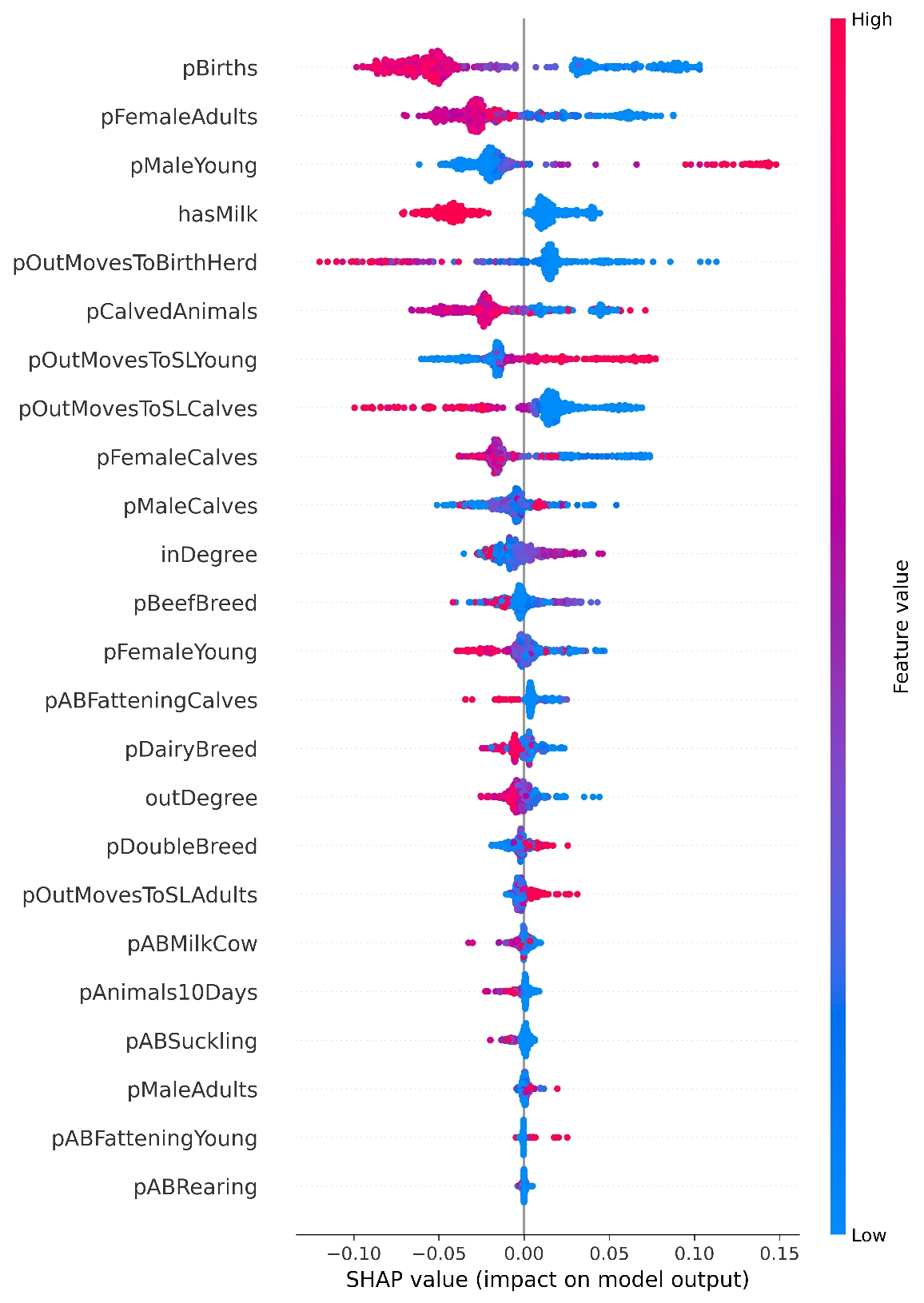


Supplementary Figure 3: Beeswarm plot of SHAP values by feature, illustrating their direction and impact on the prediction of the cattle fattening class.


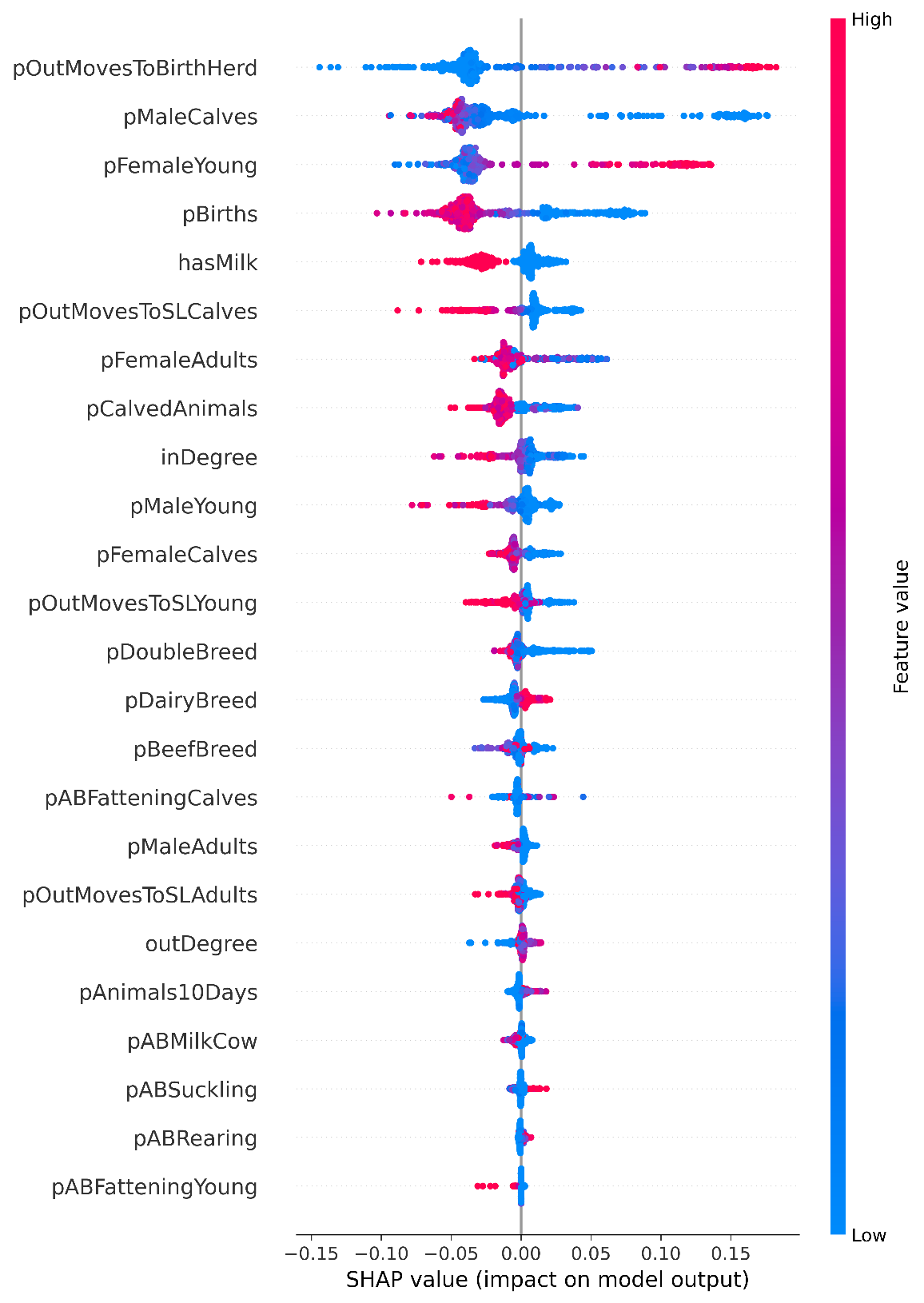


Supplementary Figure 4: Beeswarm plot of SHAP values by feature, illustrating their direction and impact on the prediction of the rearing cattle class.


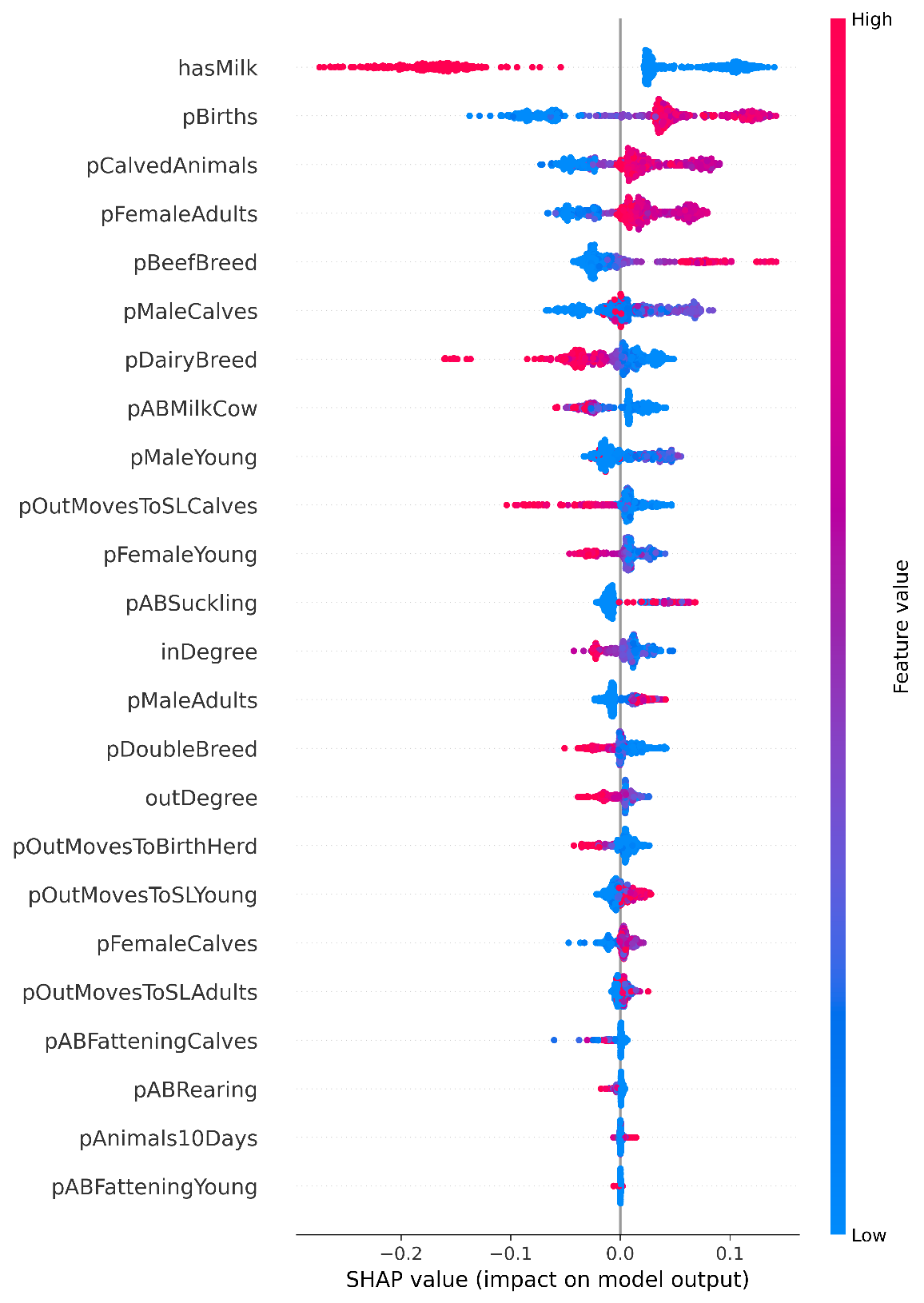


Supplementary Figure 5: Beeswarm plot of SHAP values by feature, illustrating their direction and impact on the prediction of the suckler cow class.
